# Diagnostic medical artificial intelligence: Futuristic prospects for implementation in healthcare settings

**DOI:** 10.3389/frai.2023.1169244

**Published:** 2023-03-30

**Authors:** Vishruth M. Nagam

**Affiliations:** Los Rios Community College District, Sacramento, CA, United States

**Keywords:** diagnosis, medical artificial intelligence (medical AI), implementation, patent, feasibility

## Highlights

– Medical artificial intelligence (AI) has the potential to improve the quality and efficiency of patient assessment and diagnosis.
– Here, the topics of data collection, efficiency and feasibility, and patentability are discussed in relation to the implementation of diagnostic medical AI in medical settings.
– Potential resolutions and future directions are provided to facilitate an agreeable implementation of diagnostic medical AI systems.

## Introduction

A medical facility in which a patient could instantly receive a diagnosis based on symptoms and reported concerns, where a supercomputer nurse checks the patient's vitals, might seem like a work of fiction. However, such medical care has been made an attainable possibility by artificial intelligence (AI) in medicine. Diagnostic medical AI systems function by relating known medical patterns with data and/or visual imagery of patients to formulate medical diagnoses. Though having the potential to improve the quality and efficiency of diagnoses and, ultimately, medical care, diagnostic medical AI systems must be patented and, when implemented, supervised by medical professionals and experts.

## Data collection

Because medical AI systems learn many patterns of medical conditions, they are able to diagnose otherwise relatively uncertain cases. Machine learning methods are “very sensitive to patterns emerging from small changes spread across a large number of variables” (Bruffaerts, 2018). Learning by taking vast data sets into account, diagnostic medical AI systems can identify and characterize the many different cases that may present themselves in a clinical practice. A 2017 study showed significant promise for medical AI as a diagnostic tool; the diagnostic medical AI system used in the study for early esophageal neoplasia, an early stage of cancer in the esophagus, returned an overall diagnostic accuracy of 79.38% and a sensitivity, specificity, positive predictive value, and negative predictive value of 73.41%, 83.54%, 72.09%, and 84.44%, respectively (Zhang et al., 2017).

Concerns may regard diagnostic medical AI systems as potentially limited by their very reliance on data analysis to perform and function. To maintain diagnostic accuracies above or on par with those of physicians, medical AI systems may “require large amounts of well-selected data and extensive validation,” which may potentially make them an inefficient diagnostic tool (Bruffaerts, 2018). Mitigating this concern, automated data collection techniques could be developed and implemented in medical AI systems, which would be able to then collect data and visual imagery from medical records; in such a scenario, guidelines must be developed for the safeguards and privacy of information in medical records. In order to be implemented in dynamic clinical environments and provide diagnostic services to the diverse populations of patients therein, diagnostic medical AI systems must be developed in such a way to be fast-learning and adaptable.

## Feasibility

Diagnostic medical AI may be a relatively cost- and time-efficient tool for healthcare providers and systems. The efficiency of a patient's treatment in part depends on the accuracy and quickness of a diagnosis, both of which diagnostic medical AI may be able to improve (Siuly et al., 2018). Additionally, medical AI could also suggest potential care plans, based on the nature of diagnoses, that may help quicken the process of care development by medical professionals and ultimately care delivery to patients. Scholars at Indiana University developed “an AI system that second-guessed the decisions of clinicians in 500 cases involving behavioral-health patients with a variety of other chronic illnesses”; their artificial neural network “came up with alternative, evidence-based care paths that outperformed those of the human decision makers in terms of cost while boosting patient outcomes 30 to 35%” (Conn, 2013). Additionally, artificial neural networks do not take breaks, as “they are programmed for long hours and can continuously perform without getting bored or distracted” (Siuly et al., 2018).

An alternative possibility must be considered that diagnostic medical AI systems may amount to greater, rather than lower, costs over time for providers. AI machines are complex and often incur significant manufacturing costs, which result in higher selling prices to healthcare providers and systems (Siuly et al., 2018). Additionally, once implemented, medical AI systems would regularly require and need to receive “upgradation to cater to the needs of the changing environment” and patient populations at medical facilities (Siuly et al., 2018). Minimizing the manufacturing and operational costs of diagnostic medical AI systems may improve the efficiency of their implementation in medical settings. This goal could be supported by medical AI manufacturers and developers, healthcare providers, and governments (Garvin, 2020).

## Patentability

The patentability of medical AI systems and innovations is another important consideration in this discussion. Patents are a means of incentivizing innovation of products and inventions and help spur greater knowledge dissemination and utilization of such intellectual property (Cohen et al., 2022).

AI for medical use is currently highly disputed regarding patent eligibility and moral principles. With respect to patents in the United States, the most pressing question about the patentability of medical AI systems is that of subject matter eligibility, which is a two-step test by the United States Patent and Trademark Office (USPTO) of whether the purpose of the product is “directed to a patent-ineligible concept” (laws of nature, abstract thought, etc.) and whether the new product is different enough from other patented products to be considered original (Conn, 2013; Tull, 2018). Given its aim to contribute to the operations of healthcare systems and the medical care of patients, medical AI passes the first step. However, as diagnostic medical AI systems utilizing visual data “relat[e] known images to new cases and extrapolat[e] based on the similarities or differences between the two”—similar to the manner in which physicians perform diagnostic patient examinations based on what they can see—this type and application of diagnostic medical AI has been argued as failing to pass the second step of the subject matter eligibility test (Conn, 2013; Tull, 2018). In order for diagnostic medical AI systems to be issued patents, medical AI manufacturers and developers should provide sufficient differentiation between the diagnosis methods of medical AI and those of medical professionals. Governments and legal officials should also be involved and versed in the subject matter of medical AI in order to make these important decisions on the patentability of medical AI systems.

A plausible objection may be that AI systems should not be patented because “human intelligence [shouldn't] be replicated” by AI, and machines “cannot make decisions if they encounter an [unfamiliar] situation” as do humans (Reddy, 2023). A resolution to this objection could involve supervision by a medical professional or expert while diagnostic medical AI systems are operating (Opfermann et al., 2017); in the event of disagreements or discrepancies between medical AI systems with supervisors, the supervisor could exercise their discretion to proceed in the diagnostic process. Human intelligence could take a supervisory role with respect to the implementation of diagnostic medical AI systems.

## Conclusion

The implementation of diagnostic medical AI in clinical settings is a multifaceted topic. From automated data collection systems, to cost- and time-efficiency, to patentability, a multifaceted approach involving such diverse topics should be taken to study the equitable and agreeable implementation of diagnostic medical AI systems. Future directions include analyzing patent law in additional countries and surveying stakeholders involved–including healthcare providers, medical AI manufacturers and developers, legal officials, and patient bodies–regarding the use of diagnostic medical AI systems.

## Author contributions

The author confirms being the sole contributor of this work and has approved it for publication.
